# Demographically adjusted normative data among Peruvians with diverse education levels for version 3 of the Alzheimer's Disease Centers’ neuropsychological test battery in the Uniform Data Set

**DOI:** 10.1002/alz.70671

**Published:** 2025-09-23

**Authors:** Gregory Brown, Diego Bustamante‐Paytan, María Fe Albujar Pereira, Jose Huilca, Belén Custodio, Katherine Agüero, Graciet Verastegui, Zadith Yauri, Pamela Bartolo, Daniela Bendezu, Rosa Montesinos, Nilton Custodio

**Affiliations:** ^1^ Unidad de Investigación de Deterioro Cognitivo y Prevención de Demencia Instituto Peruano de Neurociencias Lince Peru; ^2^ Department of Neurology University of California, San Francisco San Francisco California USA; ^3^ Department of Internal Medicine Medstar Washington Hospital Center Washington District of Columbia USA; ^4^ Unidad de Investigación y Docencia Equilibria Santiago de Surco Peru; ^5^ Centro de Investigación del Envejecimiento Universidad de San Martín de Porres La Molina Peru

**Keywords:** dementia, Latin America, low education, normative date, psychometrics

## Abstract

**INTRODUCTION:**

Demographically adjusted norms are essential for interpreting cognitive tests, yet none exist in Peru for the Alzheimer's Disease Centers’ (ADCs) neuropsychological test battery, especially among low‐education populations.

**METHODS:**

We assessed 340 healthy adults (ages 43–79, 70% female), balanced by education level (0–6 years, *n* = 173; ≥ 7 years, *n* = 167). Participants completed the Uniform Data Set version 3 Neuropsychological Battery (UDS3‐NB). Normative values were generated using linear regression with age, education, and sex as predictors.

**RESULTS:**

Education groups were matched for age (*p* = 0.970) and sex (*p* = 0.904). Neuropsychological measurements varied significantly between education groups, with education emerging as the primary influential factor across all measures, except semantic fluency: vegetables.

**DISCUSSION:**

This study provides comprehensive normative cognitive data for Peruvian adults across a range of education levels, which will assist in the more precise identification of cognitive impairments. Clinicians can determine individual *z* scores for each neuropsychological measure with the included calculator.

**Highlights:**

- We developed demographically adjusted normative data and a *z* score calculator for the Uniform Data Set version 3 Neuropsychological Battery among Peruvian adults.
- Recruitment was balanced by education level (0–6 vs. ≥ 7 years) to reflect the diverse educational backgrounds in Peru.
- Education was the strongest predictor of performance across most cognitive tests, particularly for working memory/executive tasks.
- These normative data support more accurate cognitive assessments and dementia diagnoses in Peruvian populations.

## 1 BACKGROUND

Latin America is experiencing a rapid demographic transition, with aging populations driving an increase in the prevalence of Alzheimer's disease (AD) and related dementias (ADRD). In Peru, the burden of cognitive disorders is rising steadily,^1^, ^2^ yet diagnostic and clinical tools remain poorly adapted to the cultural, linguistic, and educational diversity of the population.^3^, ^4^ Educational attainment in Peru varies widely, with nearly 20% of older adults having completed ≤ 6 years of formal schooling, particularly in rural and underserved urban areas.^5^ Such disparities can significantly influence neuropsychological test performance, leading to both over‐ and under‐diagnosis of cognitive impairment.

The clinical diagnosis of mild cognitive impairment (MCI) and dementia is primarily based on neuropsychological assessments. However, test performance reflects not only cognitive status but also demographic and sociocultural factors such as age, sex, education, quality of education, language, and test familiarity.^6^, ^7^ Using normative data from populations that do not share these contextual characteristics, such as norms developed in high‐income countries, can produce misleading results and reduce diagnostic accuracy.^8^ The American Academy of Clinical Neuropsychology has explicitly emphasized that the need for contextually relevant normative standards enhances diagnostic accuracy and reduces misclassification risk.^9^ Cognitive assessments have been shown to vary across countries, which challenges the ability for accurate diagnosis.^10^ Furthermore, timely identification of dementia can provide immediate impact, particularly in identifying reversible causes and enabling care planning for individuals and their families.^11^

Peru lacks comprehensive, demographically adjusted normative data for widely used neuropsychological instruments, including the Uniform Data Set version 3 Neuropsychological Battery (UDS3‐NB) developed by the National Alzheimer's Coordinating Center (NACC).^12^ As Peru has moved toward a national dementia plan, accurate and efficient diagnosis has become one of the primary pillars of improving care.^13^ One of the key advantages of the UDS3‐NB is its standardized administration protocol, developed under the guidance of the Clinical Task Force (CTF), an expert group convened by the National Institute on Aging to promote harmonized longitudinal data collection across Alzheimer's Disease Centers (ADCs). This harmonization facilitates cross‐site collaboration and comparison. Furthermore, this battery has been shown to be able to capture AD pathology.^14^

Importantly, the original developers have emphasized the need for future work to generate normative data in underrepresented populations.^12^ However, normative datasets for Peru and Latin American populations remain sparse. Existing norms for Spanish speakers often focus on United States–based Latino populations, who differ in language exposure, educational quality, and sociocultural background compared to Peruvians.^15^ The development of context‐specific reference values would provide valuable information for health‐care providers in Peru to evaluate cognitive function in their community, but these data simply do not exist.

To address this critical gap, we developed normative data for the UDS3‐NB in a sample of cognitively healthy Peruvian adults, with balanced representation across high and low education levels. Our study aims to (1) assess the influence of demographic variables (age, sex, and years of education) on cognitive test performance, and (2) provide demographically adjusted normative tables that can support more accurate and equitable detection of cognitive impairment in Peru. These data serve as a foundational step toward culturally sensitive cognitive assessment in South America and help lay the groundwork for future adaptations of dementia diagnostic criteria across the region.

## 2 METHODS

### 2.1 Participants

Participants for this cross‐sectional study were recruited between July 2020 and July 2024 at the Instituto Peruano de Neurociencias (IPN), a premier neurodegenerative center in Peru with substantial expertise in leading and managing cognitive research studies, including recruitment.^16^ Participant recruitment occurs via a well‐established referral network in Lima and through community‐based outreach and advertisements. Due to a particular interest in balanced recruitment for low‐education adults, a specific emphasis was placed on recruitment from the Villa El Salvador community. Normal cognition participants were specifically recruited by requesting individuals > 40 years and “carrying out activities normally.” All participants were evaluated in Lima, though some traveled from up to 200 km away within the greater Lima region.

Each participant completed brief cognitive and functional tests (Rowland Universal Dementia Assessment Scale [RUDAS]^17^ and Pfeffer Functional Activities Questionnaire [PFAQ])^18^ and the Clinical Dementia Rating (CDR) scale.^19^ For this study on normative data, we included participants with CDR = 0, normal functioning assessment, normal blood tests, and normal imaging, as well as consensus normal cognition based on a meeting among neurologists, geriatricians, psychiatrists, and neuropsychologists. This would include both normal functioning adults from the community and those with subjective cognitive complaints or seeking cognitive evaluation but normal comprehensive testing.

Other inclusion criteria included Spanish‐language fluency (self‐reported Spanish as the preferred language and native Spanish speaker or native indigenous language speaker with Spanish spoken as a second language > 10 years), age > 40, and functional literacy. For all participants, functional literacy was determined by self‐reporting “Are you able to read and write?” and having a normal PFAQ functional score. Participants with 0 years of education were also asked to write their name and place of birth, read and interpret the phrase “Tito plays with Dora” (Tito juega con Dora), and accurately complete the calculations 4 + 3 and 9 − 4 (¿cuánto es 4 + 3? y ¿cuánto es 9 − 4?). Exclusion criteria were: individuals with difficulty performing the cognitive tests, due to hearing, visual, or other physical problems that could interfere with their performance; individuals with a language other than Spanish; individuals with a diagnosis of dementia or MCI; participants who were diagnosed/or had symptoms compatible with psychiatric illness (bipolar disorder, psychosis, schizophrenia, and personality disorders); concomitant cerebrovascular pathology; with a history of addiction or substance abuse; and individuals with hypothyroidism, vitamin B12 deficiency, liver disease, chronic kidney disease, neuro‐infections (infection associated with human immunodeficiency virus or syphilis), severe cranioencephalic trauma, subdural hematoma, among others. We further excluded participants who in the last seven consecutive days prior to the evaluation had taken any of the following medications: opioids, decongestants, anti‐spasmodics, anti‐cholinergics, anti‐arrhythmics, anti‐depressants, anti‐psychotics, such as valproate, phenobarbital, fentanyl, carbamazepine, and levetiracetam. In cases in which patients were taking these medications for a chronic illness, and only if their medical condition would allow it, it was recommended to stop their medication for seven consecutive days prior to commencing the cognitive assessment.

**RESEARCH IN CONTEXT**

1. **Systematic review**: While the Uniform Data Set version 3 Neuropsychological Battery (UDS3‐NB) is widely used in Alzheimer's disease research, normative data have not been developed for Peruvian populations. This gap is especially critical given the high variability in education levels and limited access to culturally appropriate assessments in Latin America.
2. **Interpretation**: We present the first demographically adjusted normative data for the UDS3‐NB among Peruvian adults, including individuals with as little as 0 to 6 years of formal education. These norms enable more accurate identification of cognitive impairment in Peru and support equitable dementia diagnosis.
3. **Future directions**: Future studies should determine clinically useful thresholds for mild cognitive impairments and dementia, as well as the generalizability of these norms to other Spanish‐speaking countries in Latin America. Integrating additional culturally relevant variables such as literacy, rural versus urban residence, and quality of education could also be considered.

### 2.2 Measures

#### 2.2.1 Demographic information

Age, years of education, sex, and number of languages spoken were ascertained via a self‐reported questionnaire. Years of education were established using the question: “How many years did you go to school?” All participants indicated that Spanish was their preferred language, and the following question was also asked, “How many other languages do you speak fluently” (¿Cuántos idiomas habla fluidamente el participante en total?) and “Are any of these indigenous languages?” (¿El (la) participante habla alguna lengua indígena?).

#### 2.2.2 Neuropsychological evaluation

All assessments were performed in Spanish using the Spanish Version of the UDS3‐NB^20^ including tests assessing several cognitive domains: attention (digit span forward and backward), processing speed (Trail Making Test Part A [TMT‐A]), executive functioning (Trail Making Test Part B [TMT‐B]), naming (Multilingual Naming Test [MINT[), phonemic fluency (letters P and M for Spanish speakers), semantic fluency (animal and vegetable naming), visuospatial skills (Benson Complex Figure [BCF]‐immediate copy), and learning and memory (Craft Story 21 [CS‐21] immediate and delayed recall and BCF‐delayed recall). The Spanish adaptation of the UDS3‐NB involved preliminary translation and content review by expert clinicians, pilot testing at three US and four Latin‐American sites, including Peruvian participants, and subsequent cultural adaptations, as previously reported.^20^ All tests can be downloaded for free from the NACC website.^21^

### 2.3 Statistical analysis

All statistical analyses were conducted using MATLAB (MathWorks). Descriptive statistics were computed for demographic variables, and group comparisons between individuals with low versus high education attainment were performed to assess potential demographic differences. Independent sample *t* tests were used to compare mean age and years of education between groups, and Fisher exact test was used to compare sex distributions due to small cell sizes. Normative data were derived using linear regression models for each cognitive test score, with age (continuous, in years), education (continuous, in years), and sex (coded as 1 for male and 0 for female) included as predictors. For each test, the regression model took the form: Score_expected_ = β_intercept_ + β_Age_ (Age) + β_Education_ (Education) + β_Sex_ (Sex). From this, patient‐specific *z* score = (Score_observed_—Score_expected_)/Standard Deviation of Residuals. This method allows for the generation of demographically adjusted normative values and the quantification of individual performance relative to peers with similar demographic characteristics.

## 3 RESULTS

### 3.1 Demographics

Our study included 340 total individuals, with a mean age of 64 and 70% were female (Table 1). The cohort was balanced for low (*n* = 173) and high education (*n* = 167). The splits were similar in age (*p* = 0.970) and sex (*p* = 0.904). The low education group had a majority with 4 to 6 years of education (60%), with roughly one third < 4 years of education. In this group, 67 individuals indicated they spoke another language, which was exclusively an indigenous language (i.e., Quechua or Aymara). The higher education group primarily had 13 to 18 years of education (70%), and 32 individuals indicated they spoke another language. Of these, 21 spoke non‐indigenous languages (i.e., English), and 5 spoke more than two languages.

**TABLE 1:** Demographic characteristics.

Characteristic	Overall (*n* = 340)	Low education (*n* = 173)	High education (*n* = 167)	*p* value^a^
Age				0.970
Mean (SD)	64.1 (7.5)	64.1 (7.6)	64.1 (7.5)	
Range	43–79	43–79	43–79	
*N* (%)				
40–60	86 (25.2)	43 (24.9)	43 (25.7)	
60–70	173 (50.9)	88 (50.9)	85 (50.9)	
70–80	81 (23.8)	42 (24.3)	39 (23.4)	
Education				<0.0001
Mean (SD)	9.4 (5.8)	4.2 (1.6)	14.9 (2.7)	
Range	0 (24)	0 (6)	7 (24)	
*N* (%)				
<4	56 (16.5)	56 (32.4)	0 (0)	
4–6	117 (34.4)	117 (67.6)	0 (0)	
7–9	3 (0.8)	0 (0)	3 (1.8)	
10–12	31 (9.1)	0 (0)	31 (18.6)	
13–15	55 (16.2)	0 (0)	55 (32.9)	
16–18	67 (19.7)	0 (0)	67 (40.1)	
19+	11 (3.2)	0 (0)	11 (6.6)	
Sex, *N* (%)				0.904
M	93 (27.3)	48 (0.3)	45 (26.9)	
F	247 (72.7)	125 (0.7)	122 (73.1)	
Languages spoken, *N* (%)				<0.0001
1	241 (70.8)	106 (61.3)	135 (80.8)	
>1	99 (29.1)	67 (38.7)	32 (19.2)	

*Note*: Demographics for the total sample overall (*N* = 340) and split by low (0–6 years) and high (≥ 7 years) education.

Abbreviation: SD, standard deviation.

^a^ Results from independent sample *t* tests (for age and education) and Fisher exact test (for sex) between low and high education individuals.

### 3.2 Effects of education

The two groups varied significantly (*p* < 0.001) for all cognitive tests based on education groups (Table 2). Among age, education, and sex, education emerged as the primary influential factor across all measures, except semantic fluency: vegetables. The Cohen *d* for the effect of education ranged from 0.46 to 1.58, with 85% having a Cohen *d* > 1. The strongest effects were in TMT‐B, time (*d* = 1.58), TMT‐A, time (*d* = 1.44), Craft Story Immediate Verbatim (*d* = 1.44), and finally phonemic fluency M (*d* = 1.41) and P (*d* = 1.41). The smallest effects were semantic fluency: vegetables (*d* = 0.46) and TMT errors (for part A, *d* = 0.51, and for part B, *d* = 0.76). Non‐linear modeling (powers = 0.5, 2, 3) showed minimal improvement over linear models for age and education, typically adding ≤ 1% explained variance (Table S1 in supporting information). For education, however, non‐linear terms modestly improved model fit (5%–8%) for TMT‐A, MINT, and BCF‐immediate copy.

**TABLE 2:** UDS version 3.0 neuropsychological battery neuropsychological measure among Peruvians grouped by low (0–6 years) and high (≥ 7 years) education.

		Low education				High education		
Domain	UDS neuropsychological measure	*N*	M (SD)	Range	*N*	M (SD)	Range	*p* value^a^
Attention	Number Span Test Forward, total correct	173	3.9 (1.5)	1–9	167	6.5 (2.5)	1–14	<0.001
	Number Span Test Forward, longest span	173	4.3 (1)	2–9	165	5.7 (1.4)	3–9	<0.001
	Number Span Test Backward, total correct	170	3.1 (1.3)	0–8	167	5.3 (1.7)	2–11	<0.001
	Number Span Test Backward, longest span	171	2.9 (0.9)	0–6	167	4.1 (1)	2–7	<0.001
Processing speed	Trail Making Test Part A, time (s)	168	95.2 (34)	35–150	167	51.5 (23)	19–150	<0.001
	Trail Making Test Part A, errors	169	0.5 (0.9)	0–4	167	0.1 (0.4)	0–3	<0.001
Executive function	Trail Making Test Part B, time (s)	97	237.7 (66.1)	54–300	166	132.8 (66.8)	41–300	<0.001
	Trail Making Test Part B, errors	97	2.2 (2.2)	0–13	166	0.8 (1.1)	0–5	<0.001
Language: naming	MINT, total score	173	25.1 (4)	9–31	167	29.4 (2.1)	21–32	<0.001
Language: verbal fluency	Phonemic Fluency, M, total in 60s	173	8.2 (3.4)	1–20	167	14.2 (4.4)	2–26	<0.001
	Phonemic Fluency, P, total in 60s	173	7.2 (3.7)	0–18	167	12.8 (4.1)	2–28	<0.001
	Semantic Fluency, Animals, total in 60s	173	15.9 (4.1)	2–28	167	21.6 (5.2)	8–39	<0.001
	Semantic Fluency, Vegetable, total in 60s	173	14.9 (4.1)	4–28	167	17 (4.9)	6–35	<0.001
Visuospatial	Benson Complex Figure Copy, Immediate, total score	173	14.1 (2.1)	7–16	167	15.9 (1)	10–17	<0.001
Memory	Benson Complex Figure Copy, Delayed, total score	172	9.8 (4.5)	0–20	167	16.3 (5.9)	3–29	<0.001
	Craft Story 21, Immediate Recall, Verbatim, total units	172	7.7 (3.7)	0–16	167	13.7 (4.6)	1–25	<0.001
	Craft Story 21, Immediate Recall, Paraphrase, total units	173	8.3 (4.1)	0–16	167	12 (2.9)	3–17	<0.001
	Craft Story 21, Delayed Recall, Verbatim, total units	172	8.4 (4.7)	0–20	166	14.4 (5.7)	0–28	<0.001
	Craft Story 21, Delayed Recall, Verbatim, total units	172	7.2 (3.9)	0–23	166	12.6 (4.8)	0–22	<0.001

*Note*: Higher scores indicate better scores except for the Trail Making Test Parts A and B.

Abbreviations: MINT, Multilingual Naming Test; SD, standard deviation; UDS, Uniform Data Set.

^a^ Results from independent sample *t* tests between low and high education individuals.

### 3.3 Normative date

We can use Table 3 or the included calculator to compute the patient‐specific *z* score. Each β estimate indicates that a 1 ‐unit change in that metric causes a β estimate change in test performance. The *R* ^2^ for each model ranged from 0.077 to 0.477 (Table 3), which indicates the amount of variance attributed to these three covariates (age, education, and sex). The larger the *R* ^2^, the more important it is to correct for these factors. The TMT (Part A and Part B) had the highest explained variance, followed by phonemic fluency (M and P). Similarly, for the TMT errors and semantic fluency: vegetables had the least explained variance.

**TABLE 3:** Multiple linear regression coefficients (95% CIs) on neuropsychological test raw scores by demographic factors (age, education, sex).

UDS measure	β_Intercept_	β_Age_	β_Education_	β_Sex_	Std of resids	Rsq	Relative importance
Number Span Test Forward, total correct	4.34 (6.26, 2.42)	−0.02 (0.01, −0.05)	0.23 (0.27, 0.19)	0.02 (0.51, −0.47)	2.03	0.310	Education, age, sex
Number Span Test Forward, longest span	4.09 (5.21, 2.97)	−0.01 (0.01, −0.02)	0.13 (0.15, 0.11)	0.04 (0.33, −0.25)	1.18	0.294	Education, age, sex
Number Span Test Backward, total correct	3.88 (5.31, 2.45)	−0.02 (0, −0.04)	0.19 (0.21 ,0.16)	−0.11 (0.25, −0.48)	1.52	0.345	Education, age, sex
Number Span Test Backward, longest span	3.36 (4.26, 2.46)	−0.01 (0, −0.03)	0.11 (0.13, 0.09)	−0.07 (0.16, −0.3)	0.95	0.318	Education, age, sex
Trail Making Test Part A, time (s)	57.15 (83.39, 30.91)	0.87 (1.27, 0.47)	−3.79 (−3.28, −4.31)	−12.69 (−5.99, −19.4)	27.69	0.423	Education, age, sex
Trail Making Test Part A, errors	0.52 (1.18, −0.15)	0 (0.01, −0.01)	−0.03 (−0.02, −0.05)	−0.1 (0.07, −0.27)	0.7	0.077	Education, sex, age
Trail Making Test Part B, time (s)	157.38 (222.4, 92.32)	1.99 (2.98, 1)	−9.95 (−8.63, −11.26)	−12.06 (4.76, −28.88)	60.63	0.477	Education, age, sex
Trail Making Test Part B, errors	2.35 (4.06, 0.63)	0 (0.03, −0.02)	−0.12 (−0.09, −0.16)	0.17 (0.61, −0.28)	1.6	0.160	Education, sex, age
MINT, total score	26.73 (29.7, 23.76)	−0.05 (0, −0.09)	0.37 (0.42, 0.31)	0.74 (1.5, −0.02)	3.16	0.326	Education, age, sex
Phonemic Fluency, M, total in 60s	8.67 (12.29, 5.06)	−0.04 (0.02, −0.09)	0.53 (0.6, 0.46)	−0.77 (0.15, −1.69)	3.84	0.399	Education, sex, age
Phonemic Fluency, P, total in 60s	8.73 (12.26, 5.21)	−0.05 (0, −0.1)	0.51 (0.57, 0.44)	−1.2 (−0.3, −2.1)	3.74	0.397	Education, sex, age
Semantic Fluency, Animals, total in 60s	21.75 (26, 17.5)	−0.12 (−0.06, −0.19)	0.51 (0.6, 0.43)	−0.24 (0.84, −1.33)	4.51	0.329	Education, age, sex
Semantic Fluency, Vegetable, total in 60s	19.93 (23.97, 15.89)	−0.07 (−0.01, −0.13)	0.16 (0.24, 0.08)	−3.14 (−2.1, −4.17)	4.29	0.154	Sex, education, age
Benson Complex Figure Copy, Immediate, total score	15.14 (16.66, 13.63)	−0.03 (−0.01, −0.05)	0.16 (0.19, 0.14)	0.47 (0.86, 0.08)	1.6	0.282	Education, age, sex
Benson Complex Figure Copy, Delayed, total score	13 (16.31, 9.69)	−0.1 (−0.05, −0.15)	0.31 (0.38, 0.25)	1.09 (1.93, 0.24)	3.51	0.250	Education, age, sex
Craft Story 21, Immediate Recall, Verbatim, total units	8.3 (13.19, 3.42)	−0.01 (0.06, −0.08)	0.59 (0.68, 0.49)	−0.73 (0.52, −1.97)	5.18	0.306	Education, sex, age
Craft Story 21, Immediate Recall, Paraphrase, total units	7.48 (11.38, 3.58)	−0.03 (0.03, −0.09)	0.53 (0.6, 0.45)	−0.25 (0.75, −1.25)	4.13	0.358	Education, age, sex
Craft Story 21, Delayed Recall, Verbatim, total units	9.54 (14.47, 4.6)	−0.05 (0.03, −0.12)	0.52 (0.62, 0.42)	−0.51 (0.75, −1.77)	5.21	0.258	Education, age, sex
Craft Story 21, Delayed Recall, Verbatim, total units	6.61 (10.8, 2.43)	−0.02 (0.05, −0.08)	0.46 (0.54, 0.38)	−0.14 (0.93, −1.21)	4.41	0.268	Education, age, sex

*Note*: Higher scores indicate better scores except for the Trail Making Test Parts A and B, so must take the negative of the patient‐specific *z* score for Trail Making Test Parts A and B. Patient‐specific *z* score = (Score_observed_—Score_expected_)/Standard Deviation of Residuals, where Score_expected_ = β_intercept_ + β_Age_ (Age) + β_Education_ (Education) + β_Sex_ (Sex). Age and Education are in years. Sex = 1 for male and Sex = 0 for female.

Abbreviations: CI, confidence interval; MINT, Multilingual Naming Test; Std of Resids, standard deviation of residuals; UDS, Uniform Data Set.

## 4 DISCUSSION

### 4.1 Main findings

This study presents demographically adjusted normative data for the NACC UDS3‐NB in a Peruvian population, addressing a critical gap in culturally appropriate cognitive assessment tools for Latin America. Normative data were derived using linear regression models that included age, sex, and education, allowing for demographic correction in cognitive test interpretation. Importantly, our sample included > 170 individuals with 0 to 6 years of education, a demographic that remains significantly underrepresented in global cognitive research. This inclusion is especially relevant given that education had the strongest association with cognitive performance of the three demographic variables. These data support health‐care providers in making accurate cognitive evaluations and contribute to broader international efforts, led by initiatives such as the NACC, to harmonize neuropsychological tools and establish equitable, culturally valid standards for dementia diagnosis and research worldwide.

Studies conducted in Latin America and the Caribbean (LAC) have found that educational attainment is a stronger dementia risk factor compared to genetic ancestry,^22^ and a systematic review of literature from 17 LAC countries found that those without formal education had double the risk of dementia (21.4%) compared to those with at least 1 year of formal education (9.9%).^23^ Furthermore, individuals in LAC countries may display a higher brain age (based on functional magnetic resonance imaging and electroencephalography) compared to non‐LAC countries,^24^ which may be due to structural socioeconomic inequality, pollution, and health disparities. Notably, the LAC countries exhibit a lower education level than the United States, and these disparities may play a crucial role in shaping geographical differences in gray matter volume and connectivity.^25^ However, all of these studies included populations with > 6 years of education. More studies are needed to investigate cognitive impairment in individuals with < 6 years of education, and this will only be possible with rigorous normative data that enable consistent dementia diagnoses. Existing harmonization frameworks outline common standards for normative data collection, including diagnostic consensus panels, appropriate age ranges, and sufficient sample sizes.^26^ Our data align with these principles, supporting utility in establishing reproducible and rigorous dementia research for low‐education, Spanish‐speaking adults. Context‐specific normative scores, particularly from under‐represented groups such as low‐education adults, could establish appropriate cutoffs (usually *z* scores of −1.5 or −2) and provide insights into specific risk factors, imaging changes, blood biomarkers, or treatment strategies.^27^ Valid classification of cognitive status is a prerequisite for accurately investigating factors that modulate cognitive decline in low‐ versus high‐education individuals.

We observed particularly strong effect sizes of education for executive attention and verbal memory. Bosma et al. also reported lower education was associated with more rapid decline in processing speed and verbal memory.^28^ However, many studies have reported the lack of association between education and specific cognitive domains.^29^ Instead, education is more viewed as a passive cognitive reserve.^30^ Low education is a risk factor for dementia,^31^ but our findings, based on a rigorously screened sample of cognitively healthy individuals, suggest that some studies may overestimate dementia prevalence in low‐education populations due to the use of cutoffs that do not adequately account for education background. Furthermore, education is only one demographic factor that may relate to cognitive test performance; we found age was associated with processing speed, verbal fluency, and memory, which is consistent with other normative studies,^15^, ^32^ and aligns with known age‐related trajectories in cognitive decline.^33^ We found sex to be the most important factor for semantic fluency: vegetables, which was also found in a study of demographic normative data for Latino individuals from the United States.^15^ Some studies report sex‐ but not education‐related differences in semantic fluency,^34^ but the reasons for this remain an open area of investigation. Impairments in semantic fluency are well documented in AD and are often attributed to degradation of semantic networks.^35^, ^36^ This impairment has been linked to hypometabolism in temporal lobe structures involved in conceptual processing, such as the inferior temporal gyrus.^37^ More work is needed to understand the mechanisms behind demographic influence on cognitive domains.

Clinically, these normative tables offer a robust method for estimating individualized *z* scores based on a patient's demographic profile, using a simple formula:

$$\mathrm{Patient}\;z\;\mathrm{score}=\frac{\mathrm{Scor}e_{\mathrm{observed}}-\mathrm{Scor}e_{\mathrm{expected}}}{\mathrm{Standard}\;\mathrm{Deviation}\;\mathrm{of}\;\mathrm{Residuals}}$$

where Score_expected_ = β_intercept_ + β_Age_ ∗ (Age) + β_Education_ ∗ (Education) + β_Sex_ ∗ (Sex). Age and Education are in years. Sex = 1 for male and Sex = 0 for female. We have also supplied a calculator in the supporting information to facilitate computation of scores. This supports more contextually accurate cognitive assessments, especially critical for detecting early signs of ADRD in populations traditionally underrepresented in neuropsychological research. Dementia diagnosis has been a key initiative of national dementia plans, and our findings advance diagnostic capabilities in Peru.^38^ Furthermore, these data may be useful for any individual with a low education level, as there are very little normative data for individuals with < 6 years of education, and standardized cognitive tools risk misclassifying normal variation as pathological. However, this requires further validation.

### 4.2 Limitations

Several limitations warrant consideration. First, while our sample was balanced by education and sex, it was limited to an urban population, potentially reducing generalizability to rural areas where education disparities and dialectical variations may be more pronounced. Second, our models did not include literacy, socioeconomic status, or quality of education, factors known to influence test performance and cognitive reserve. Third, our oldest adult was 79 years of age, so future studies should extend to adults 80+ years of age, who are at the highest risk for dementia. Finally, low‐education individuals in our study disproportionately spoke an indigenous language as well. More investigations are needed to understand the complex interaction of multilingualism and cognitive testing.

## 5 CONCLUSION

This study provides the first comprehensive, demographically stratified normative cognitive data for Peruvian adults, which will assist in more precise identification of cognitive impairments. The generated tables can be used to determine individual *z* scores and are valid for all education levels in Peruvian individuals ranging from 43 to 79 years of age. Further validation of these norms in clinical practice is needed to identify thresholds for MCI and dementia. Also, studies should assess the generalizability of these norms across other Spanish‐speaking countries in Latin America, where sociocultural and educational contexts differ. Additionally, integrating culturally relevant variables such as rural versus urban residence, multilingualism, literacy, and school quality may further refine normative accuracy. Ultimately, these norms lay the foundation for culturally grounded cognitive assessment in Peru and may serve as a critical stepping‐stone toward more equitable and accurate dementia diagnosis across Latin America, advancing both local clinical care and global health equity in cognitive aging.

## CONFLICT OF INTEREST STATEMENT

The authors declare that they have no conflicts of interest related to the research, authorship, and/or publication of this article. Author disclosures are available in the Supporting Information.

## CONSENT STATEMENT

All participants participated voluntarily in the study and provided written informed consent.

## Supporting information



Supporting Information

Supporting Information

Supporting Information

## References

[1] Custodio N , García A , Montesinos R , Escobar J , Bendezú L . Prevalencia de demencia en una población urbana de Lima‐Perú: estudio puerta a puerta. An Fac Med. 2013;69(4):233. doi:10.15381/anales.v69i4.1110

[2] Herrera‐Perez E , Custodio N , Diaz M , et al. Epidemiology of neurocognitive disorders in adults from urban‐marginalized areas: a door‐to‐door population‐based study in Puente Piedra, Lima, Peru. Front Public Health. 2023;11:1228008. doi:10.3389/fpubh.2023.1228008 37927880 PMC10622761

[3] Custodio N , Wheelock A , Thumala D , Slachevsky A . Dementia in Latin America: epidemiological evidence and implications for public policy. Front Aging Neurosci. 2017;9:221. doi:10.3389/fnagi.2017.00221 28751861 PMC5508025

[4] Parra MA , Baez S , Allegri R , et al. Dementia in Latin America: assessing the present and envisioning the future. Neurology. 2018;90(5):222‐231. doi:10.1212/WNL.0000000000004897 29305437 PMC5791795

[5] Cueto S . Height, weight, and education achievement in rural Peru. Food Nutr Bull. 2005;26(2 Suppl 2):S251‐S260. doi:10.1177/15648265050262s216 16075575

[6] Ardila A . Cultural values underlying psychometric cognitive testing. Neuropsychol Rev. 2005;15(4):185‐195. doi:10.1007/s11065-005-9180-y 16395623

[7] Merkley TL , Esopenko C , Zizak VS , et al. Challenges and opportunities for harmonization of cross‐cultural neuropsychological data. Neuropsychology. 2023;37(3):237‐246. doi:10.1037/neu0000818 35549387

[8] Czerwinski‐Alley NC , Chithiramohan T , Subramaniam H , Beishon L , Mukaetova‐Ladinska EB . The effect of translation and cultural adaptations on diagnostic accuracy and test performance in dementia cognitive screening tools: a systematic review. J Alzheimers Dis Rep. 2024;8(1):659‐675. doi:10.3233/ADR-230198 38746627 PMC11091753

[9] American Academy of Clinical Neuropsychology . American Academy of Clinical Neuropsychology (AACN) practice guidelines for neuropsychological assessment and consultation. Clin Neuropsychol. 2007;21(2):209‐231. doi:10.1080/13825580601025932 17455014

[10] Nichols E , Ng DK , Hayat S , et al. Differences in the measurement of cognition for the assessment of dementia across geographic contexts: recommendations for cross‐national research. Alzheimers Dement. 2023;19(3):1009‐1019. doi:10.1002/alz.12740 35841625 PMC9891734

[11] Power MC , Willens V , Prather C , et al. Risks and benefits of clinical diagnosis around the time of dementia onset. Gerontol Geriatr Med. 2023;9:23337214231213185. doi:10.1177/23337214231213185 PMC10666707 38026091

[12] Weintraub S , Besser L , Dodge HH , et al. Version 3 of the Alzheimer Disease Centers’ Neuropsychological Test Battery in the Uniform Data Set (UDS). Alzheimer Dis Assoc Disord. 2018;32(1):10‐17. doi:10.1097/WAD.0000000000000223 29240561 PMC5821520

[13] Diaz MM , Pintado‐Caipa M , Garcia PJ . Challenges in implementation of public policies in aging and dementia in Peru. PLOS Glob Public Health. 2023;3(9):e0002345. doi:10.1371/journal.pgph.0002345 37676846 PMC10484423

[14] Čihák M , Horáková H , Vyhnálek M , et al. Evaluation of differential diagnostics potential of Uniform Data Set 2 neuropsychology battery using Alzheimer's disease biomarkers. Arch Clin Neuropsychol. 2024;39(7):839‐848. doi:10.1093/arclin/acae028 38582748 PMC11504696

[15] Marquine MJ , Parks A , Perales‐Puchalt J , et al. Demographically‐adjusted normative data among Latinos for the version 3 of the Alzheimer's Disease Centers’ Neuropsychological Test Battery in the Uniform Data Set. Alzheimers Dement. 2023;19(9):4174‐4186. doi:10.1002/alz.13313 37356069 PMC10622863

[16] Custodio B , Mora‐Pinzon M , Montesinos R , et al. Improving participants’ recruitment in dementia‐related studies on social media through colloquial language in Lima, Peru. Dement Neuropsychol. 2025;19:e20240232. doi:10.1590/1980-5764-dn-2024-0232 40469243 PMC12136589

[17] Custodio N , Montesinos R , Lira D , et al. Validation of the RUDAS in patients with a middle‐level education in Lima, Peru. Am J Alzheimers Dis Dementias. 2019;34(7‐8):513‐522. doi:10.1177/1533317519869709 PMC10653366 31422688

[18] Quiroga LP , Albala BC , Klaasen PG . Validación de un test de tamizaje para el diagnóstico de demencia asociada a edad, en Chile. Rev Médica Chile. 2004;132(4). doi:10.4067/s0034-98872004000400009 15382519

[19] Hughes CP , Berg L , Danziger W , Coben LA , Martin RL . A new clinical scale for the staging of dementia. Br J Psychiatry. 1982;140(6):566‐572. doi:10.1192/bjp.140.6.566 7104545

[20] Besser L , Kukull W , Knopman DS , et al. Version 3 of the National Alzheimer's Coordinating Center's Uniform Data Set. Alzheimer Dis Assoc Disord. 2018;32(4):351‐358. doi:10.1097/WAD.0000000000000279 30376508 PMC6249084

[21] Uniform Data Set version 3. National Alzheimer's Coordinating Center. Accessed July 28, 2025. https://naccdata.org/data‐collection/forms‐documentation/uds‐3

[22] Llibre‐Guerra JJ , Jiang M , Acosta I , et al. Social determinants of health but not global genetic ancestry predict dementia prevalence in Latin America. Alzheimers Dement. 2024;20(7):4828‐4840. doi:10.1002/alz.14041 38837526 PMC11247688

[23] Ribeiro F , Teixeira‐Santos AC , Caramelli P , Leist AK . Prevalence of dementia in Latin America and Caribbean countries: systematic review and meta‐analyses exploring age, sex, rurality, and education as possible determinants. Ageing Res Rev. 2022;81:101703. doi:10.1016/j.arr.2022.101703 35931410 PMC9582196

[24] Moguilner S , Baez S , Hernandez H , et al. Brain clocks capture diversity and disparities in aging and dementia across geographically diverse populations. Nat Med. 2024;30(12):3646‐3657. doi:10.1038/s41591-024-03209-x 39187698 PMC11645278

[25] Gonzalez‐Gomez R , Legaz A , Moguilner S , et al. Educational disparities in brain health and dementia across Latin America and the United States. Alzheimers Dement. 2024;20(9):5912‐5925. doi:10.1002/alz.14085 39136296 PMC11497666

[26] Boccardi M , Monsch AU , Ferrari C , et al. Harmonizing neuropsychological assessment for mild neurocognitive disorders in Europe. Alzheimers Dement. 2022;18(1):29‐42. doi:10.1002/alz.12365 33984176 PMC9642857

[27] Dodge HH , Goldstein FC , Wakim NI , et al. Differentiating among stages of cognitive impairment in aging: version 3 of the Uniform Data Set (UDS) neuropsychological test battery and MoCA index scores. Alzheimers Dement Transl Res Clin Interv. 2020;6(1):e12103. doi:10.1002/trc2.12103 PMC7683960 33283037

[28] Bosma H , Van Boxtel MPJ , Ponds RWHM , Houx PJ , Burdorf A , Jolles J . Mental work demands protect against cognitive impairment: MAAS prospective cohort study. Exp Aging Res. 2003;29(1):33‐45. doi:10.1080/03610730303710 12735080

[29] Lenehan ME , Summers MJ , Saunders NL , Summers JJ , Vickers JC . Relationship between education and age‐related cognitive decline: a review of recent research. Psychogeriatrics. 2015;15(2):154‐162. doi:10.1111/psyg.12083 25516261

[30] Christensen H , Korten AE , Jorm AF , et al. Education and decline in cognitive performance: Compensatory but not protective. Int J Geriatr Psychiatry. 1997;12(3):323‐330. doi:10.1002/(SICI)1099-1166(199703)12:3<323::AID-GPS492>3.0.CO;2-N 9152716

[31] Sharp ES , Gatz M . Relationship between education and dementia: an updated systematic review. Alzheimer Dis Assoc Disord. 2011;25(4):289‐304. doi:10.1097/WAD.0b013e318211c83c 21750453 PMC3193875

[32] Sachs BC , Steenland K , Zhao L , et al. Expanded demographic norms for version 3 of the Alzheimer Disease Centers’ Neuropsychological Test Battery in the Uniform Data Set. Alzheimer Dis Assoc Disord. 2020;34(3):191‐197. doi:10.1097/WAD.0000000000000388 32483017 PMC7842186

[33] Salthouse TA . Selective review of cognitive aging. J Int Neuropsychol Soc. 2010;16(5):754‐760. doi:10.1017/S1355617710000706 20673381 PMC3637655

[34] Cameron RM , Wambaugh JL , Mauszycki S . Effects of age, gender, and education on semantic fluency for living and artifact categories. Aphasiology. 2008;22(7‐8):790‐801. doi:10.1080/02687030701818018

[35] Verma M , Howard RJ . Semantic memory and language dysfunction in early Alzheimer's disease: a review. Int J Geriatr Psychiatry. 2012;27(12):1209‐1217. doi:10.1002/gps.3766 22298328

[36] Weintraub S , Wicklund AH , Salmon DP . The neuropsychological profile of Alzheimer disease. Cold Spring Harb Perspect Med. 2012;2(4):a006171‐a006171. doi:10.1101/cshperspect.a006171 22474609 PMC3312395

[37] Melrose RJ , Campa OM , Harwood DG , Osato S , Mandelkern MA , Sultzer DL . The neural correlates of naming and fluency deficits in Alzheimer's disease: an FDG‐PET study. Int J Geriatr Psychiatry. 2009;24(8):885‐893. doi:10.1002/gps.2229 19296551 PMC2743128

[38] Hampel H , Vergallo A , Iwatsubo T , et al. Evaluation of major national dementia policies and health‐care system preparedness for early medical action and implementation. Alzheimers Dement. 2022;18(10):1993‐2002. doi:10.1002/alz.12655 35293672 PMC9790361

