# Supplementary material for: Demographically adjusted normative data among Peruvians with diverse education levels for version 3 of the Alzheimer's Disease Centers’ neuropsychological test battery in the Uniform Data Set
Source: Alzheimers Dement. 2025 Sep 23;21(9):e70671. doi: 10.1002/alz.70671 (PMC12457076; doi:10.1002/alz.70671)
Supplement: Supplementary file 1 — Supporting Information [file ALZ-21-e70671-s003.docx]

**Supplemental Table 1. Exploration on Non-linear Effects of Age and Education on UDS3 Tests**

|  | **Age** | | | **Education** | | |
| --- | --- | --- | --- | --- | --- | --- |
| **UDS measure** | Linear R^2^ | Best Order | R^2^ Gain over linear | Linear R^2^ | Best Order | R^2^ Gain over linear |
| Number Span Test Forward, total correct | 0.0076 | 0.5 | 0.0049 | 0.3053 | 0.5 | 0.0150 |
| Number Span Test Forward, longest span | 0.0021 | 0.5 | 0.0031 | 0.2927 | 0.5 | 0.0098 |
| Number Span Test Backward, total correct | 0.0133 | 0.5 | 0.0005 | 0.3355 | 0.5 | 0.0134 |
| Number Span Test Backward, longest span | 0.0130 | 3 | 0.0005 | 0.3087 | 0.5 | 0.0031 |
| Trail Making Test Part A, time (s) | 0.0326 | 3 | 0.0048 | 0.3716 | 0.5 | 0.0530 |
| Trail Making Test Part A, errors | 0.0009 | 0.5 | 0.0067 | 0.0725 | 3 | 0.0141 |
| Trail Making Test Part B, time (s) | 0.0303 | 3 | 0.0033 | 0.4438 | 0.5 | 0.0075 |
| Trail Making Test Part B, errors | 0.0006 | 3 | 0.0091 | 0.1580 | 0.5 | 0.0223 |
| MINT, total score | 0.0121 | 0.5 | 0.0265 | 0.3110 | 0.5 | 0.0858 |
| Phonemic Fluency, M, total in 60s | 0.0071 | 0.5 | 0.0023 | 0.3909 | 3 | 0.0066 |
| Phonemic Fluency, P, total in 60s | 0.0127 | 3 | 0.0011 | 0.3772 | 3 | 0.0137 |
| Semantic Fluency, Animals, total in 60s | 0.0356 | 0.5 | 0.0069 | 0.3007 | 3 | 0.0032 |
| Semantic Fluency, Vegetable, total in 60s | 0.0238 | 3 | 0.0021 | 0.0437 | 0.5 | 0.0093 |
| Benson Complex Figure Copy, Immediate, total score | 0.0147 | 3 | 0.0146 | 0.2594 | 0.5 | 0.0541 |
| Benson Complex Figure Copy, Delayed, total score | 0.0338 | 3 | 0.0071 | 0.2082 | 3 | 0.0114 |
| Craft Story 21, Immediate Recall, Verbatim, total units | 0.0013 | 3 | 0.0072 | 0.3029 | 3 | 0.0081 |
| Craft Story 21, Immediate Recall, Paraphrase, total units | 0.0039 | 3 | 0.0053 | 0.3555 | 3 | 0.0130 |
| Craft Story 21, Delayed Recall, Verbatim, total units | 0.0067 | 3 | 0.0050 | 0.2531 | 3 | 0.0121 |
| Craft Story 21, Delayed Recall, Verbatim, total units | 0.0021 | 0.5 | 0.0165 | 0.2671 | 3 | 0.0145 |

Abbreviations: MINT: Multilingual Naming Test; UDS: Uniform Data Set.

Associations of fitting polynomials with the form y=ax^p^ +b where x is the Age or Education, y is the UDS3 test scores, p=0.5, 1, 2, and 3. Model fit with least-squares regression. The linear R^2^ is presented and the best fit with the improvement over the linear model is presented. Sex is a binary variable (Male=1, Female=0), so nonlinear associations would be irrelevant.
